# In-depth sequence analysis of highly-conserved *pyrH* gene to study distributions of oral treponemes in periodontal disease versus health

**DOI:** 10.1080/20002297.2017.1325210

**Published:** 2017-06-01

**Authors:** Rory M. Watt

**Affiliations:** ^a^ Faculty of Dentistry, University of Hong Kong, Hong Kong, China

## Abstract

**Background:** More than 75 species/species-level phylotypes of oral treponeme bacteria inhabit the oral cavity. However, their respective genomic compositions and clinical distributions remain poorly understood.

**Objectives:** To compare distributions of phylogroup 1 and 2 oral treponemes in subjects with various periodontal health conditions, via sequence analysis of a highly-conserved treponeme ‘housekeeping’ gene.

**Methods:** Subgingival plaque samples were collected from Chinese subjects with chronic periodontitis (n=5), aggressive periodontitis (n=4), gingivitis (n=5), and healthy controls (n=4). Samples were analyzed by a PCR/plasmid clone sequencing-based approach, using primer sets targeting the *pyrH* gene. Data was analyzed using various computational/bioinformatic approaches.

**Results:** 1,227 quality-filtered *pyrH* gene sequences were obtained (mean 66.2±9.6 sequences per subject), which were assigned to 33 ‘*pyrH* genotypes’ (97% identity cut-off). 538 *pyrH* sequences (17 *pyrH* genotypes) corresponded to phylogroup 1 treponemes (including ‘*T. vincentii*’, *Treponema medium*, and ‘*Treponema sinensis*’ taxa). 689 *pyrH* sequences (16 *pyrH* genotypes) corresponded to phylogroup 2 taxa. Correlations between *pyrH* genotype distributions and disease status were complex. One *pyrH* genotype, which was phylogenetically-related to *T. denticola* GM-1/MS25 strains, was highly prevalent: being detected in 17/18 subjects.

**Conclusions:** Both healthy and periodontally-diseased subjects harbor multiple genetic lineages corresponding to the same treponeme species/phylotype within their subgingival niches.

